# Clinico-pathological considerations in a 48-years-old female with acute kidney injury: is it lupus nephritis, ANCA-associated vasculitis or something else?

**DOI:** 10.1186/s12882-019-1531-7

**Published:** 2019-08-27

**Authors:** Marie Lemerle, Anne-Sophie Garnier, Anne Croue, Alain Chevailler, Jean-Paul Saint-André, Jean-François Subra, Jean-François Augusto, Julien Demiselle

**Affiliations:** 1 Service de Néphrologie-Dialyse-Transplantation, Université d’Angers, CHU Angers, 4 rue Larrey, 49033 Angers CEDEX 09, Angers, France; 2 LUNAM Université, Angers, France; 3 Département de Pathologie Cellulaire et Tissulaire, Université Angers, CHU Angers, Angers, France; 4 Laboratoire d’Immunologie, Université d’Angers, CHU Angers, Angers, France

**Keywords:** Lupus glomerulonephritis, ANCA, Immunology

## Abstract

**Background:**

The value of ANCA positivity in the setting of systemic lupus erythematous and their pathogenicity remains uncertain.

**Case presentation:**

We report the case of a 48-year-old female with rapidly progressive kidney failure, arthro-myalgia and weight loss. Auto-immune screening showed anti-dsDNA antibodies, complement consumption and triple ANCA positivity. A first kidney biopsy done at presentation highlighted class IV-G glomerulonephritis with elective extra-capillary involvement and mainly C1q glomerular deposition at immunofluorescence study. After three months of a regimen combining steroids and cyclophosphamide, a second biopsy was performed and showed class IV-G glomerulonephritis with mainly endocapillary proliferation.

**Conclusion:**

This case is atypical in view of immunological profile and kidney histopathological presentation and evolution and gives rise to discussion in view of recent data on ANCA value in lupus nephritis, and suggests that different auto-immune pathways may be involved in lupus nephritis.

## Background

Several lines of evidence indicate that anti-neutrophil cytoplasmic antibodies (ANCAs), especially myeloperoxidase (MPO)-ANCAs, are pathogenic auto-antibodies in ANCAs-associated vasculitis (AAV) [1]. However, ANCAs can also be detected in healthy subjects and in a number of inflammatory/auto-immune diseases where their pathophysiological significance remains debatable [2].

Detection of ANCAs at diagnosis or in the course of systemic lupus erythematosus (SLE) is quite common, reported in up to 20% of patients [3]. According to literature, perinuclear ANCAs (p-ANCAs) rather than cytoplasmic ANCAs (c-ANCAs) are detected using indirect immunofluorescence (IIF) in SLE [3, 4]. However, the significance of these ANCAs is quite variable among SLE patients, some of them having ANCAs directed towards minor ANCA antigens (ie, lactoferrin, BPI, elastase.), and others towards major ANCA antigens (MPO or PR3), the latters’ being those associated with AAV [3]. Even if ANCA positivity has been assoiated with higher disease activity in SLE patients, their potential role in organ injury still have to be demonstrated [3].

Recently, Tuner-Stokes et al. studied retrospectively more than 200 kidney biopsies from patients with lupus nephritis (LN) and compared histopathological features of those associated with ANCAs at biopsy to those without [4]. As already reported in past studies, a predominance of p-ANCA of MPO specificity was observed in SLE patients [3]. More interestingly, they found that patients with ANCA positivity had more frequently diffuse glomerulonephritis with segmental involvement (class IV-S LN ISN/RPS classification) and lesions of glomerular necrosis, as compared to patients without ANCAs. Moreover, ANCA positive patients had a worse kidney function at the time of biopsy, higher anti-dsDNA antibody levels and lower complement levels. Thus, ANCAs positivity was associated with a specific kidney histological and biological phenotype in SLE patients. These observations allow to reopen an old debate on the potential pathogenic action of ANCAs in LN [5].

Here, we report the case of a 48-years old female referred to our department with systemic symptoms and acute kidney injury. The clinical and biological presentation was very suggestive of SLE, but strong ANCA positivity was also detected. The kidney histology at admission and its evolution 4 months after treatment initiation was uncommon and gives rise to discussion.

## Case presentation

A 48-years old Asian female was referred to our hospital with a recent history of weakness, myalgia and arthralgia. She also complained of anorexia with 3 kg weight loss. She had no past medical history and was free of any medication on admission. At presentation, blood pressure was 180/80 mmHg, heart rate was 91/min, temperature was normal. Heart and lung auscultation, as well as abdominal examination, were normal. She had no skin involvement nor lymphadenopathy or synovitis.

Biology showed acute kidney injury with serum creatinine at 209 μmol/L. White blood cell count (4.8 G/L) and platelet count (269 G/L) were normal, but anemia with hemoglobin at 7.8 g/dL was present. C-reactive protein was slightly increased (14 mg/dL). Urinalysis revealed glomerular proteinuria (proteinuria to creatinine ratio (P/C) 3.6 g/g) and microscopic hematuria. Kidney ultrasound examination showed normal sized kidneys and excluded obstruction. Thus, we concluded to acute glomerular syndrome and performed immunological laboratory tests. Antinuclear antibodies (ANA) (1/2560), as well as anti-dsDNA (292 UI/mL), anti-SSA and anti-SSB antibodies were detected. Type 3 cryoglobulinemia and complement consumption (decreased C3, C4 and CH50) were also present. Search for lupus anticoagulant, IgG anti-cardiolipin and anti-beta-2GP1 antibodies was negative. Finally, pANCA were detected at 1/2000 titer using IIF. ELISA showed the concomitant presence of MPO, PR3 and lactoferrine ANCAs with a strong positivity for MPO and lactoferrine ANCAs and a low positivity for PR3 ANCAs. ANCA detection using multiplex technology also detected MPO ANCA at high level, and low PR3 ANCA level.

**Fig. 1:**
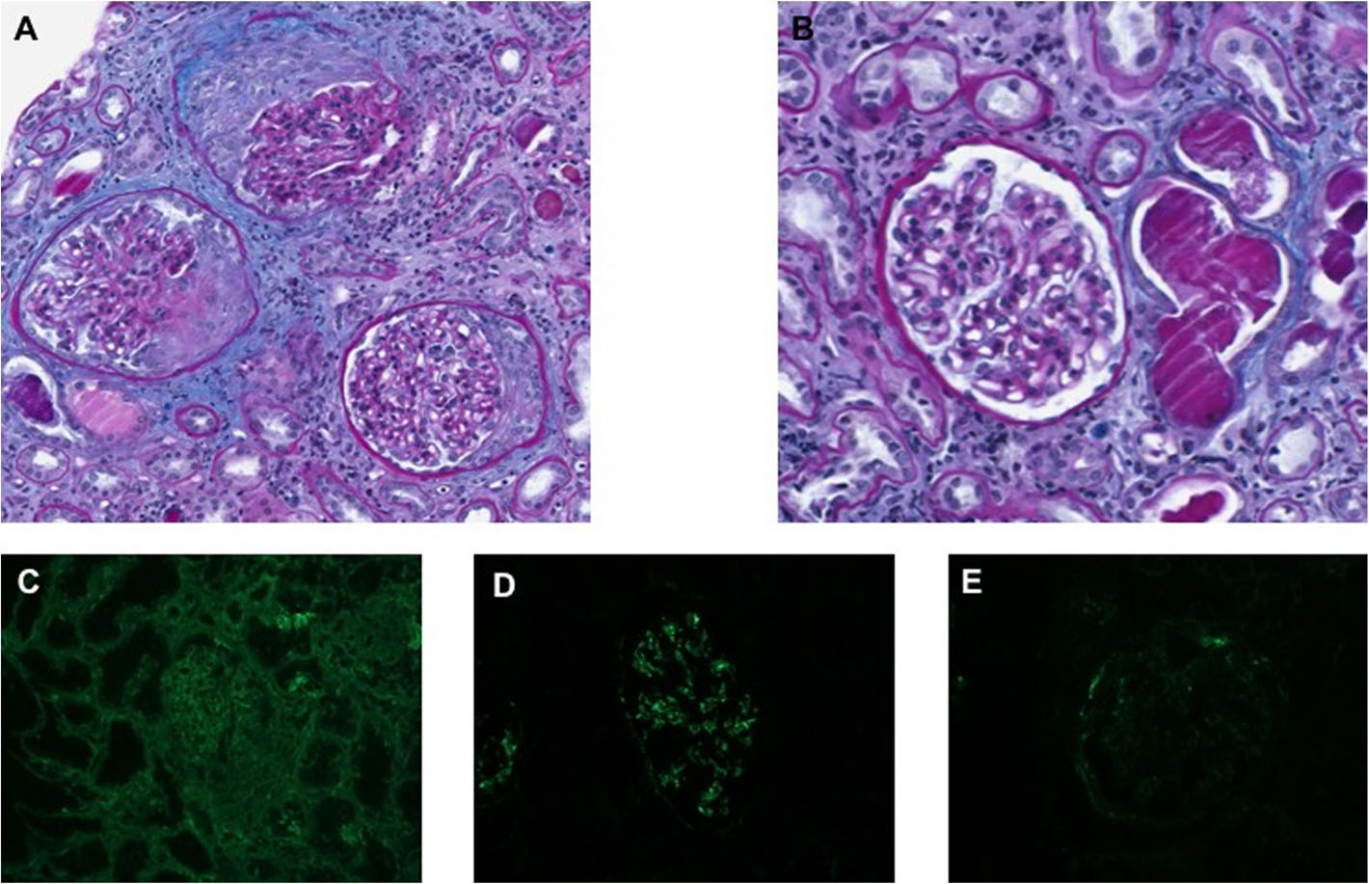
Kidney biopsy done at admission. (**a**,**b**) Light microscopy examination showing (**a**) cellular circumferential crescents within 3 glomeruli, and (**b**) a normal glomeruli. Periodic acid-Schiff staining. (**c**, **d**, **e**), Immunofluorescence analysis showing predominant C1q staining (**d**), as compared to IgG (**c**) and C3 (**e**) staining

LN was first considered leading us to perform a kidney biopsy. On optical examination, extra-capillary circumferential cellular or fibro-cellular crescents were observed in 10 of 16 glomeruli. Segmental endocapillary proliferation was absent to very modest, without any lesions of capillary necrosis (Fig. 1). Immunofluorescence analysis showed strong and diffuse mesangial and parietal C1q staining with a granular deposition pattern, while IgG, IgA, IgM, and C3 deposits were quite limited, of mesangial topography and only segmental (Fig. 1).

Following biopsy, an immunosuppressive treatment was initiated with an association of steroids, hydoxychloroquine and pulse intravenous cyclophosphamide (CYC, 500 mg every two weeks) according to the Euro-Lupus protocol trial [6]. Under this regimen, a progressive improvement of both her general condition and kidney function was observed. At month 3 from treatment initiation (after the 6th CYC injection), serum creatinine was 116 μmol/L and P/C ratio decreased to 0.50 g/g. Antinuclear antibodies decreased to 1/200, anti-dsDNA antibodies became undetectable, and complement returned within normal range. ANCAs were still detectable although at lower titer using IIF (1/200), with only MPO ANCAs remaining slightly positives at ELISA and multiplex assays.

**Fig. 2:**
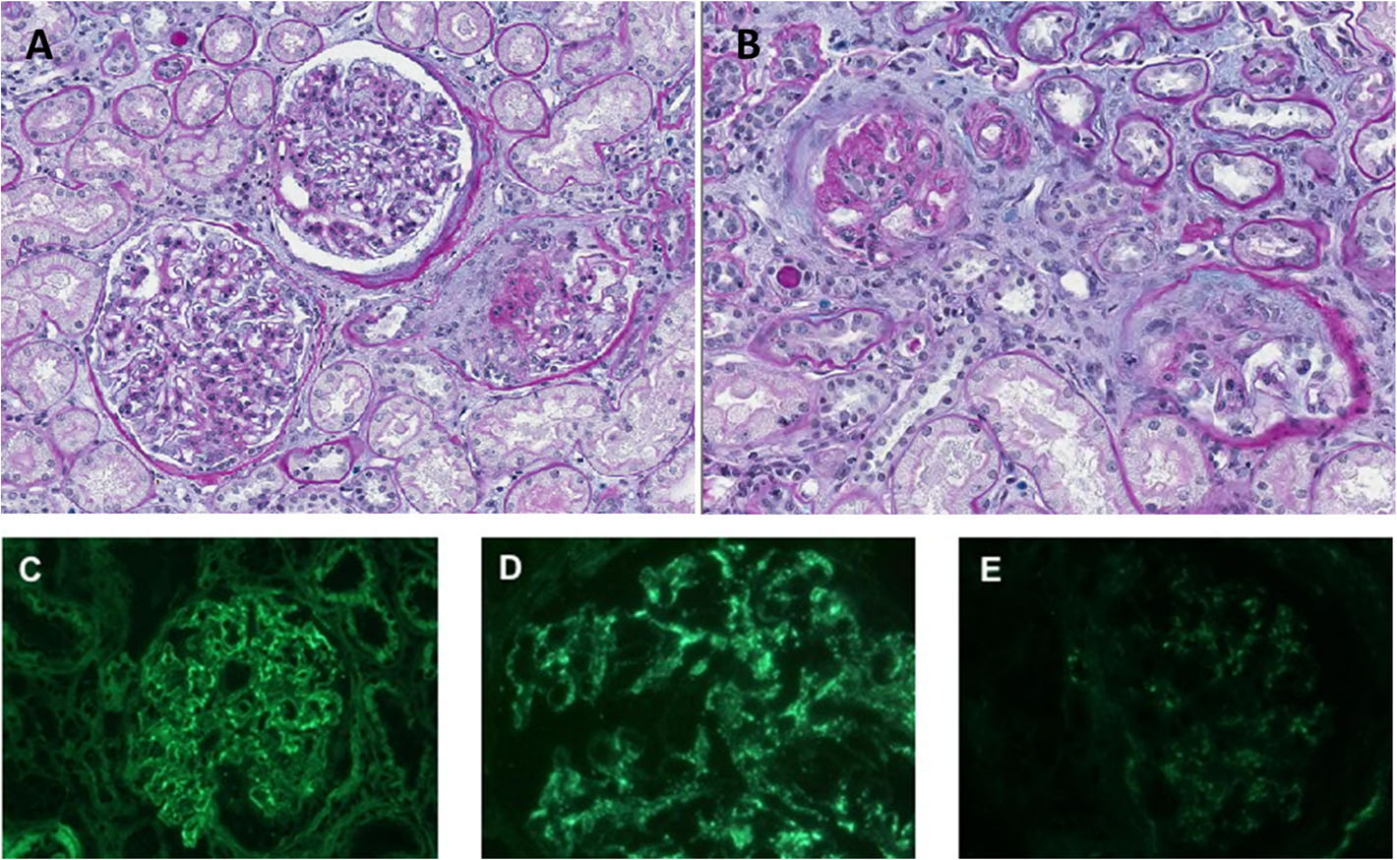
Control kidney biopsy done at month 4 from admission. (**a**,**b**) Light microscopy examination showing (**a**) diffuse endocapillary proliferation in 2 glomeruli, and (**b**) fibrotic crescents in 2 glomeruli. Periodic acid-Schiff staining staining. (**c**, **d**, **e**), Immunofluorescence analysis showing predominant C1q staining (**d**), as compared to IgG (**c**) and C3 (**e**) staining

At that time, we decided to perform a systematic kidney biopsy to analyze histological response to treatment. On optical examination, only fibrotic crescents were observed in 9/14 glomeruli, none of them being cellular of fibro-cellular. Global lesions of endocapillary proliferation were observed in most glomeruli, without lesions of capillary necrosis (Fig. 2). Immunofluorescence analysis showed diffuse mesangial and parietal C1q staining at a lower intensity as compared to the diagnostic biopsy. IgG, IgA, IgM, and C3 deposits remained limited, in their pattern and intensity (Fig. 2).

After the biopsy results, we decided to continue CYC. However, after the 8th CYC injection, the patient developed toxidermia which we attributed to CYC. At that time, we decided to start mycophenolate mofetil (MMF) at 1.5 g/day.

One year after initial admission and under MMF for 12 months, she has no clinical manifestation of SLE and did not relapse nephritis. Renal function returned to near normal values (serum creatinine of 90 μmol/L, MDRD eGFR 60 mL/min/1.73m^2^). ANCA detection was negative on IIF evaluation and ELISA, with anti-MPO being still detectable at very low level using multiplex assay. ANA were stable at 1/200, complement was in normal range and search for cryoglobulin was negative.

## Discussion and conclusion

The present case represents a very uncommon overlap presentation of SLE and AAV according to both its immunological and kidney pathological aspects. First, our patient presented with a typical SLE serology, but had also circulating auto-antibodies towards three different ANCA antigens, which is an extremely rare condition. Second, the kidney pathological lesions observed at diagnosis, as well as their evolution on control biopsy, were uncommon in comparison to those usually observed in proliferative forms of LN [7, 8]. The present case illustrates the complexity of immune mechanisms acting in SLE, and raises the question of the importance of histology findings in the choice of treatment in such situations.

The first atypical aspect in this observation is the coexistence of a typical SLE serology (ANA, anti-dsDNA ab, complement consumption) and ANCA positivity. As mentionned above, ANCAs, mainly p-ANCAs of MPO specificity, are detected in up to 20% of SLE patients, which allows to conclude that it is finally a quite common condition. However, in our patient, ANCAs directed towards three different ANCA antigens, namely MPO, PR3 and lactoferrin, were detected. According to the persistence of p-ANCA pattern while anti-dsDNA disappeared, a cross reactivity anti-dsDNA was unlikely [9]. This condition represents a quite exceptional situation that may be related to particular auto-immune pathways acting in our patient. In contrast with double positive ANCAs, directed towards a major ANCA antigen (MPO or PR3) and towards a minor ANCA antigen, that is finally frequently observed (notably in AAV), ANCA positivity towards both MPO and PR3 appears to be extremely rare [10, 11]. Indeed, in a past study, only 23 patients with this phenotype among 3095 consecutive ANCA positive patients, representing less than 1% of patients [12]. Interestingly, most patients with this condition had inflammatory or auto-immune diseases including SLE, but none had AAV. Confirming the rarity of this association, in a recent study analyzing ANCA positivity value in LN patients, double MPO/PR3 positivity was observed in 3/32 patients with ANCA positivity among 254 consecutives LN patients, thus randomly 1% of LN patients [4].

The second atypical aspect of our observation is kidney histopathology at both first and second biopsy. According to the International Society of Nephrology (ISN) / Renal Pathology Society (RPS), the first renal biopsy of our patient responded to the definition of class IV (diffuse, lesions involving > 50% of glomeruli), subtype class IV-G (global, lesions affecting > 50% of the glomerular tuft) [7]. Among class IV-G LN, crescentic forms involving more than 50% of glomeruli, where crescents take up over half space in Bowman’s capsule, have been poorly analyzed. Yu et al. observed that these crescentic forms represented 20% of 152 class IV-G LN consecutive patients [13]. Interestingly, as compared to non-crescentic one’s, all patients with crescentic class IV-G LN forms presented with rapidly progressive glomerulonephritis (RPGN) and had a poorer renal survival, with higher rates of relapse at 4 years from initial diagnosis. Moreover, while endocapillary proliferation was comparable between groups, crescentic forms were associated with significantly lesser immune complex deposition at immunofluorescence study. ANCA positivity (mainly MPO ANCAs) at biopsy was present in 30% of patients with crescentic form, which was significantly higher as compared to the 2.5% observed in patients with non-crescentic class IV-G LN. These observations show that SLE patients with ANCAs are more likely to present with crescentic LN and thus suggest a role for ANCAs in crescent development. As compared to the study of Yu et al., two particular aspects deserve to be emphasized in our case. First, endocapillary proliferation was only very limited in our patient. Second, immunofluorescence study showed an unbalanced deposition of immune complexes. Indeed, strong and diffuse C1q deposition was present, in contrast to other immune complexes that were mildly represented with only segmental deposition. It is tempting to link this atypical LN presentation to ANCA positivity by suggesting a direct pathogenic role of ANCAs or the involvement of specific auto-immune pathways related to the uncommon tolerance breakdown toward 3 different ANCA antigens observed in our patient.

This overlap between AAV and LN suggests common pathophysiological aspects. One of them could be related to Neutrophil Extracelular Traps (NETs) formation, with a different manner between AAV and LN in NETs formation (lytic versus non lytic formation) [14].

The analysis of the second biopsy is also of great interest. Indeed, all crescents became fibrotic, showing the efficiency of immunosuppressive treatment on the extra-capillary lesions. On immunofluorescence evaluation, immune complex deposits were comparable to what was observed on the first biopsy. However, and surprisingly, the endocapillary proliferation became diffuse and global, thus much more pronounced as compared to the first biopsy. It allowed to classify it into IV-G subtype, but no longer because of crescentic involvement. Finally, the present case report showed conversion from crescentic class IV-G to endocapillary proliferative class IV-G. Thus, it shows that lesions can vary between crescentic and endocapillary proliferation forms in the same patient and we suggest that this may be related to modulation of involved pathogenic pathways under immunosuppressive treatment. Interestingly, such observation of transitions between LN classes have been previously reported, especially between class IV-G and IV-S [15]. Biological determinants of these changes remain to be elucidated.

The understanding of biological pathways modulating the histological phenotype of LN may translate into more targeted and efficient therapies. In our patient, given the predominant crescentic presentation at first biopsy, we decided to initiate CYC rather than MMF as induction regimen. We speculated that, even if no fibrinoid necrosis was observed, maybe pathways common to those involved in AAV were acting. We observed a good response of the crescentic lesions to steroid/CYC regimen. Next, at the second biopsy showing mainly endocapillary proliferation, given CYC-related toxidermia, we opted rather to MMF while we were considering to start rituximab before biopsy as used in maintenance treatment of AAV. However, we must acknowledge that all these considerations remain speculatives because some reports have also suggested a better improvement of crescentic LN forms under MMF as compared to CYC [16].

This atypical case report highlights the complexity of LN pathophysiology. Transition from crescentic to endocapillary proliferative forms, which has to our knowledge not been reported previously, suggests that different auto-immune pathways may be involved at different extend and at different timings in the same patient. It also highlights the importance of more systematic kidney biopsy to better understand the pathophysiology of LN, especially in patients with atypical histological and immunological presentations as it was the case in our patient.

## Data Availability

Not applicable.

## Abbreviations

AAV: ANCA associated vasculitis
ANA: Antinuclear antibodies
ANCA: Antineutrophil cytoplasmic antibodies
AntidsDNA: Anti double strain DNA antibody
CYC: Cyclophosphamide
ISN: International Society of Nephrology
LN: Lupus nephritis
MMF: Mycophenolate Mofetil
MPO: Myeloperoxydase
NETs: Neutrophil Extracellular Traps
PR3: Proteinase 3
RPGN: Rapidly progressive glomerulonephritis
RPS: Renal Pathology Society
SLE: systemic lupus erythematosus
